# Applied Behavior Analysis Techniques for Preventing Problematic Behaviors in Psychiatric Inpatient Settings: An Evidence-Based Framework

**DOI:** 10.1192/j.eurpsy.2026.10975

**Published:** 2026-07-31

**Authors:** D. Ocaklik Altunkiliç

**Affiliations:** Faculty of Health Sciences, Yeditepe University, Istanbul, Türkiye

## Abstract

**Introduction:**

Problematic behaviors—such as aggression, agitation, self-harm, and treatment refusal—in psychiatric inpatient units significantly compromise patient safety, therapeutic alliance, and overall care quality.Applied Behavior Analysis (ABA) offers function-based,proactive interventions (including DRA/DRO,NCR,FCT,token economies,and contingency management) supported by an emerging and diverse empirical evidence base.

**Objectives:**

To synthesize high-quality evidence on ABA-based strategies for preventing problematic behaviors in psychiatric settings and to present a practical, ethically grounded clinical implementation roadmap.

**Methods:**

A targeted review of systematic reviews,randomized controlled trials, and clinical guidelines published between 2000 and 2024 was conducted. Included studies focused on functional behavior assessment, positive behavior support planning, reinforcement-based strategies, token economies, and contingency management in psychiatric populations.

**Results:**

Evidence suggests that ABA-based strategies can reduce the use of seclusion and restraint by up to 40% in inpatient units with comprehensive staff training (VA Evidence Synthesis Program 2024;APA 2020). Positive behavior support planning in adolescent units led to a 35–50% decrease in disruptive incidents (Reynolds et al.2016). Token economy interventions demonstrated improvements in social participation (mean increase of 25% in group therapy attendance) and a reduction in negative symptoms in schizophrenia populations (Dickerson et al. 2005; McMonagle & Sultana 2000). Contingency management achieved abstinence rates of 60–70% in patients with comorbid stimulant use disorders (McDonell et al. 2013). DRA/DRO and NCR produced consistent, statistically significant reductions in problem behaviors across inpatient and developmental disability contexts (Carr et al. 2000;Athens et al. 2016), while FCT interventions maintained gains for up to 6 months post-intervention (Ghaemmaghami & Hanley 2021).

**Conclusions:**

Function-based ABA interventions,when integrated into psychiatric care protocols with adequate staff training,ongoing fidelity monitoring,and ethical oversight,represent a viable and preventive alternative to restrictive practices. These interventions not only reduce the frequency and severity of problematic behaviors but also improve patient engagement, therapeutic relationships, and long-term behavioral stability. For maximal impact, psychiatric services should establish structured ABA-based prevention programs, embed function-based assessment into routine care, and allocate resources for continuous data monitoring. Future high-quality RCTs, especially in adult inpatient settings, are essential to further validate these approaches and optimize their implementation.

**Disclosure of Interest:**

None Declared

